# Decentralizing the delivery of HIV pre-exposure prophylaxis (PrEP) through family physicians and sexual health clinic nurses: a dissemination and implementation study protocol

**DOI:** 10.1186/s12913-018-3324-2

**Published:** 2018-07-03

**Authors:** Malika Sharma, Allison Chris, Arlene Chan, David C. Knox, James Wilton, Owen McEwen, Sharmistha Mishra, Daniel Grace, Tim Rogers, Ahmed M. Bayoumi, John Maxwell, Rita Shahin, Isaac Bogoch, Mark Gilbert, Darrell H. S. Tan

**Affiliations:** 1 Division of Infectious Diseases, St. Michael’s Hospital, Toronto, Canada; 2 Department of Medicine, University of Toronto, Toronto, Canada; 3 Maple Leaf Medical Clinic, Toronto, Canada; 4 Toronto Public Health, Toronto, Canada; 5 Scarborough Sexual Health Clinic, Toronto, Canada; 6 Ontario HIV Treatment Network, Toronto, Canada; 7 Gay Men’s Sexual Health Alliance, Toronto, Canada; 8 Center for Urban Health Solutions, St. Michael’s Hospital, Toronto, Canada; 9 Institute of Health Policy, Management and Evaluation, University of Toronto, Toronto, Canada; 10 Institute of Medical Sciences, University of Toronto, Toronto, Canada; 11 Dalla Lana School of Public Health, University of Toronto, Toronto, Canada; 12 Canadian Treatment Information Exchange (CATIE), Toronto, Canada; 13 Division of General Internal Medicine, St. Michael’s Hospital, Toronto, Canada; 14 AIDS Committee of Toronto, Toronto, Canada; 15 Division of Infectious Diseases, University Health Network, Toronto, Canada; 16 British Columbia Center for Disease Control, Vancouver, Canada

**Keywords:** HIV pre-exposure prophylaxis, Task shifting, Implementation science, Knowledge translation, HIV prevention, Men who have sex with men, Medical education, Task shifting

## Abstract

**Background:**

Gay, bisexual and other men who have sex with men (gbMSM) in Canada continue to experience high rates of incident HIV. Pre-exposure prophylaxis (PrEP, the regular use of anti-HIV medication) reduces HIV acquisition and could reduce incidence. However, there are too few physicians with expertise in HIV care to meet the projected demand for PrEP. To meet demand and achieve greater public health impact, PrEP delivery could be ‘decentralized’ by incorporating it into front-line prevention services provided by family physicians (FPs) and sexual health clinic nurses.

**Methods:**

This PrEP decentralization project will use two strategies. The first is an innovative knowledge dissemination approach called ‘Patient-Initiated CME’ (PICME), which aims to empower individuals to connect their family doctors with online, evidence-based, continuing medical education (CME) on PrEP. After learning about the project through community agencies or social/sexual networking applications, gbMSM interested in PrEP will use a uniquely coded card to access an online information module that includes coaching on how to discuss their HIV risk with their FP. They can provide their physician a link to the accredited CME module using the same card. The second strategy involves a pilot implementation program, in which gbMSM who do not have a FP may bring the card to designated sexual health clinics where trained nurses can deliver PrEP under a medical directive. These approaches will be evaluated through quantitative and qualitative methods, including: questionnaires administered to patients and physicians at baseline and at six months; focus groups with patients, FPs, and sexual health clinic staff; and review of sexual health clinic charts. The primary objective is to quantify the uptake of PrEP achieved using each decentralization strategy. Secondary objectives include a) characterizing barriers and facilitators to PrEP uptake for each strategy, b) assessing fidelity to core components of PrEP delivery within each strategy, c) measuring patient-reported outcomes including satisfaction with clinician-patient relationships, and d) conducting a preliminary costing analysis.

**Discussion:**

This study will assess the feasibility of a novel strategy for disseminating knowledge about evidence-based clinical interventions, and inform future strategies for scale-up of an underutilized HIV prevention tool.

## Background

### PrEP and HIV prevention

Despite decades of prevention efforts centered around behaviour change and condom use, Canada continues to see high rates of HIV acquisition, with approximately 2000 new infections a year since 2002 [1]. A disproportionate burden of infections occurs among gay, bisexual and other men who have sex with men (gbMSM), who make up 49.3% of incident HIV infections and have a 131-fold higher risk of HIV than other Canadian men [2]. The high cost of HIV treatment and care [3], the young age of those newly diagnosed (mean age 35.8) [4], and the stigma associated with HIV [5, 6] underscore the economic and social importance of preventing new infections.

To address these challenges, there is increasing interest in HIV pre-exposure prophylaxis (PrEP) as part of a combination approach to controlling the epidemic, together with behavioural, psychosocial and other biomedical approaches. PrEP refers to regular use of tenofovir disoproxil fumarate and emtricitabine (TDF/FTC) by uninfected persons at ongoing HIV risk to prevent HIV acquisition. Clinical trials and observational studies in gbMSM and transgender women show that daily PrEP decreases HIV risk by up to 99% when adherence is high [7–10]. Daily oral TDF/FTC was licensed for use as PrEP in the United States in July 2012, and approved for this indication by Health Canada in February 2016.

While PrEP is not yet used widely in Canada, several factors suggest that PrEP availability and demand are poised to increase dramatically, especially following recent regulatory approval. Interest in PrEP rose from 33.3% in 2010 to 52.5% in 2015 among gbMSM receiving anonymous HIV testing at a Toronto STI clinic [11–14]. In addition, an expert advisory committee has recommended reimbursement of PrEP by public drug plans, which would facilitate wider access [15]. Furthermore, the availability of cost-reduced generic versions of TDF/FTC has greatly increased access to PrEP, although universal coverage has not been achieved.

### Current limitations in PrEP provision

The potential increase in PrEP demand offers a timely opportunity to determine how best to deliver PrEP at scale to at-risk populations. In Canada, PrEP has mainly been prescribed in a ‘centralized’ fashion, by a limited number of specialist physicians with expertise in antiretroviral medications and HIV care. However, there are not nearly enough such physicians to meet the anticipated demand for PrEP. For example, in the first 48 h of recruiting gbMSM into PREPARATORY-5, Canada’s only PrEP demonstration project, study coordinators received 92 referrals for the project’s 50 slots [16].

Physician knowledge about PrEP remains low in Canada [17]. In 2013, a Canadian survey of infectious diseases (ID), HIV primary care, public health and internal medicine physicians demonstrated that 81% of respondents felt it should be possible for PrEP to be prescribed by any doctor, yet only 46.8% felt they themselves had enough knowledge to make informed prescribing decisions [17]. A similar study of North American ID physicians identified “not knowing enough about” PrEP as a reason for not prescribing PrEP [18]. Although PrEP knowledge has been disseminated to community members through community-based agencies, to date the only systematic effort to disseminate knowledge about PrEP to Canadian family doctors has been the publication of a Canadian guideline in November 2017 [19]. In addition, although PrEP could be provided by existing HIV specialists, reliance on specialists is costly and less accessible. A feasible and sustainable approach to decentralized PrEP delivery may help re-allocate HIV specialist time to the care for persons living with HIV and improve primary care provider capacity.

To sustainably and efficiently deliver PrEP to high-risk gbMSM at the scale needed to achieve public health impact, we posit that PrEP delivery should be decentralized by harnessing the skills of family physicians (FPs) and sexual health clinic nurses. As a first step towards this vision, this study will determine the feasibility of two corresponding strategies, each aimed at adapting PrEP delivery to these different providers and healthcare settings, as part of a unique patient-initiated approach.

### Engaging family physicians (FPs) in PrEP delivery

PrEP, like other evidence-based primary prevention strategies against chronic diseases, fits within the scope of primary care practice. Surveys of HIV specialists and non-specialists suggest that most providers feel primary care is the appropriate setting for PrEP delivery [20]. A few qualitative studies provide insights into how to achieve the goal of improving capacity among FPs to prescribe PrEP. In interviews with New York City FPs and HIV specialists, a key concern was the risk of “patient mismanagement” by FPs due to inadequate knowledge, implying that PrEP training materials must be rigorously developed and that monitoring of fidelity to the core components of PrEP delivery is crucial [20]. Evidence also suggests that some FPs may have only limited comfort and skill to discuss sexual activity, which is essential to appropriately prescribe PrEP [20, 21]. Literature on physician behaviour shows that clinicians’ motivations to learn about and integrate a new intervention into their practice are greatest when the request comes directly from one of their patients or when there is professional guidance, such as guidelines from normative bodies [21, 22].

While PrEP users may be identified by their providers, public health units using STI data, or by patients themselves, experience suggests that thus far, most individuals initiating PrEP in Canada have been self-identified. In part this is a function of rising knowledge and interest in PrEP at the community level. By encouraging patients to use standardized, self-completed screening tools as a catalyst for discussing their HIV prevention needs with their providers, this project capitalizes on the current situation by harnessing these large patient numbers to disseminate information about PrEP to providers.

### Engaging sexual health clinic nurses in PrEP delivery

Ontario’s Ministry of Health and Long-Term Care mandates that public health units provide services to reduce the burden of sexually transmitted infections (STIs) and blood-borne infections, including HIV [23]. Public health units operationalize this mandate by running sexual health clinics staffed primarily by nurses, a model replicated in most of Canada. These clinics already offer a slate of biomedical and behavioural HIV prevention interventions like counseling and STI management. PrEP delivery fits squarely within the mandate of such clinics. PrEP delivery can be readily managed by trained nurses in these settings for several reasons. PrEP has a favourable tolerability and toxicity profile, most users lack comorbidities that would necessitate specialist care and PrEP follow-up can be highly protocolized [24]. Most concomitant issues addressed during PrEP clinic visits relate to public health nurses’ existing expertise, including STIs, vaccinations, and safer sex counseling. Further, this delivery setting may be more acceptable than HIV specialists’ offices since it is community-based, and may alleviate concerns about being misconstrued as an ‘HIV patient’. In Ontario, as in other Canadian jurisdictions, physicians may delegate clinical tasks to other trained providers under a medical directive [25]. Labour market costs for nurses are less than for specialist physicians, and nurses are routinely involved in decentralized strategies for post-exposure prophylaxis (PEP) [26]. Furthermore, many individuals may be unwilling to approach their FP about their sexual health, as Canadian data suggests that nearly 50% of gbMSM are not “out” to their FPs, rendering it vital to offer alternative venues to access sexual health interventions like PrEP [27].

## Methods

### Study aim

The goal of this study is to gather preliminary feasibility and implementation outcome data on two complementary strategies for decentralizing PrEP delivery to Toronto gbMSM. These strategies involve:

1. a *dissemination intervention*, where patients are empowered to link their FPs with online continuing medical education (CME) about PrEP using an innovative ‘Patient-Initiated CME’ (PICME) approach to knowledge translation, as well as
2. *an implementation intervention*, where ‘nurse-led PrEP’ will be piloted in two sexual health clinics operated by Toronto public health under a medical directive.

### Objectives

**Fig. 1:**
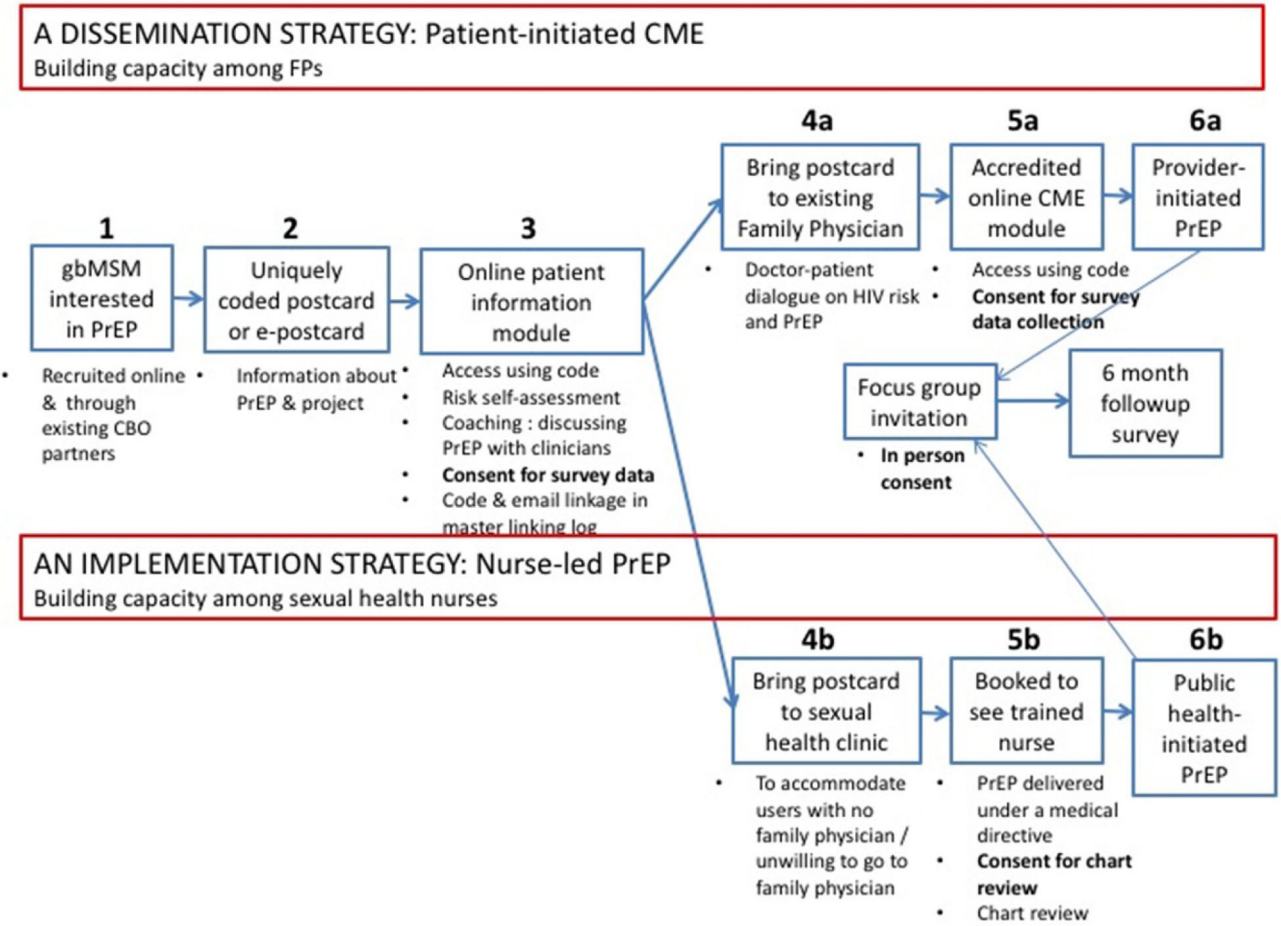
Project Overview

Our primary objective is to quantify the uptake of PrEP achieved among Toronto gbMSM using each decentralization strategy, defined as the number of patients initiated on PrEP by family physicians through the PICME approach, and by nurses in the sexual health clinics, respectively. As depicted in Fig. 1, and as described further below, each strategy incorporates several steps that must be completed between the moment when an interested gbMSM first considers using PrEP, and the moment that he initiates it through an FP or public health nurse (ie. “uptake”). We will estimate the level of PrEP uptake as a proportion of the initial number of gbMSM who initiate the process. To characterize how this cascade of events occurs, we will further quantify each step in the cascade for each strategy as an absolute number and as a proportion of the number who completed the previous event in the cascade.

Our secondary objectives include:

1. To characterize the barriers and facilitators to PrEP uptake achieved by each decentralization strategy
2. To assess fidelity to core components of PrEP delivery within each decentralization strategy as well as mediators of fidelity including accurate knowledge about PrEP among providers. Core components of PrEP delivery include a) identifying a clinical indication for PrEP at each visit, b) ascertainment of HIV negative status prior to each prescription, c) correct prescription of daily oral TDF/FTC, d) monitoring for renal and bone toxicity, and e) STI screening, based on existing guidelines [19].
3. To measure patient-reported outcomes including satisfaction with clinician-patient relationship

### Intervention

This project includes multiple components, as outlined in Fig. 1. These are best understood using a step-wise approach. In the first step, a gbMSM becomes interested in PrEP through one of two mechanisms. First, we have built a network of 23 community-based organization (CBO) partners in the Toronto area serving a wide cross-section of gbMSM [28]. These partners will disseminate information cards about the project to their clients. Second, we will post an electronic version of the information card on social/sexual networking apps. Both strategies were successfully used in the past to recruit to our PrEP trial [16].

In the second step, a potential participant obtains a uniquely coded information card about the project. The card will contain a link to an online patient module on one side, and a link to an accredited CME physician module on the reverse. The unique code will be required for both patients and providers to access the modules, permitting tracking of card distribution and linking of patient/provider responses.

The third step involves completion of the online patient information module, which will describe PrEP, the PICME principle, and project objectives in accessible language, and contain an HIV risk self-assessment tool based on validated HIV risk indices that can be used to catalyze discussions with their clinicians [29, 30]. The module will also coach users on discussing sexual behaviour and HIV risk with clinicians. This content has been developed in partnership with local gbMSM organizations, using principles of patient engagement and agency over one’s own health [31]. Further, because lack of medication coverage is an important potential barrier to PrEP uptake in this population, contact information for an existing ‘PrEP access counsellor’ at a major local AIDS service organization who can assist gbMSM in identifying and navigating drug insurance options will be provided within the module. Finally, the module includes a baseline questionnaire about demographics, sexual behaviour, and patient-provider relationship characteristics.

Fourth, patients with an existing FP will bring the information card to an appointment and use it to initiate a discussion about HIV risk and the appropriateness of using PrEP. In step 5a, physicians may then use the unique code to access the 60-min CME module on PrEP. This module was specifically developed by our team to train FPs unfamiliar with PrEP to provide appropriate PrEP care. A systematic review has demonstrated that 77% of internet-based CME interventions across a variety of domains were found to improve or maintain physician performance [22]. The web-based CME module format minimizes costs, fosters sustainability and matches the preferences of our target audience: in our nationwide survey of physicians, 84.1% indicated that online modules were a preferred format for learning more about PrEP [17]. The module will include a short survey regarding practice demographics, PrEP knowledge and beliefs, and HIV-associated stigma, as there is evidence to suggest that individuals seeking PrEP face stigma pertaining to sexual behaviours, practices, and HIV risk factors [32].

To help users assess mastery of the clinical material, we have included an end-of-module quiz based on best practices in assessment and evaluation. The literature suggests using case-based multiple choice and short answer formats related to the core components of the PrEP intervention are more indicative of performance than perceived self-assessment [33]. In addition, case-based learning can be authentic as more high-fidelity forms of simulation in medical education, and multiple-choice question formats have been shown to be predictive of clinicians’ performance in practice [34–36]. End-of-module quiz performance will be recorded.

To accommodate users who do not have a FP or are not comfortable approaching their FP despite the coaching within the patient module, users may alternatively bring their coded information postcards to one of two sexual health clinics operated by Toronto Public Health. Nurses at each site will be trained to deliver PrEP under a medical directive through in-person teaching sessions, supplemented by the same evidence-based CME modules used for family physicians. Informed consent for the prospective collection of clinic data will be sought from patients at their first visit, and the unique ID codes will allow linkage with patient data.

In the final step, it is hoped that the FP or public health nurse will initiate PrEP if indicated. We will track this outcome by triangulating data from electronic patient and provider questionnaires at baseline and at 6-months, and from TPH charts.

### Study setting

This study will take place in the greater Toronto area, an urban setting with a heavy concentration of CBOs serving gbMSM. The estimated prevalence of HIV among gbMSM in Toronto is 23% [37, 38].

### Eligibility

Participants for this study will be recruited through advertisements on a social/sexual networking applications as well as a network of 23 CBOs, where staff will distribute information cards to gbMSM clients. CBOs will be asked to specifically target individuals meeting the following criteria: identify as gbMSM, are believed to be HIV-uninfected, are interested in or appropriate for PrEP (in their own opinion or in the opinion of the CBO staff), and are a resident in the Greater Toronto Area. Participants will need to be able to understand English to complete the online modules.

### Timeline

Online modules will be launched in September 2017, with data collection taking place over the subsequent 18 months (12 months accrual and final survey at 6 months).

### Sample size considerations

**Fig. 2:**
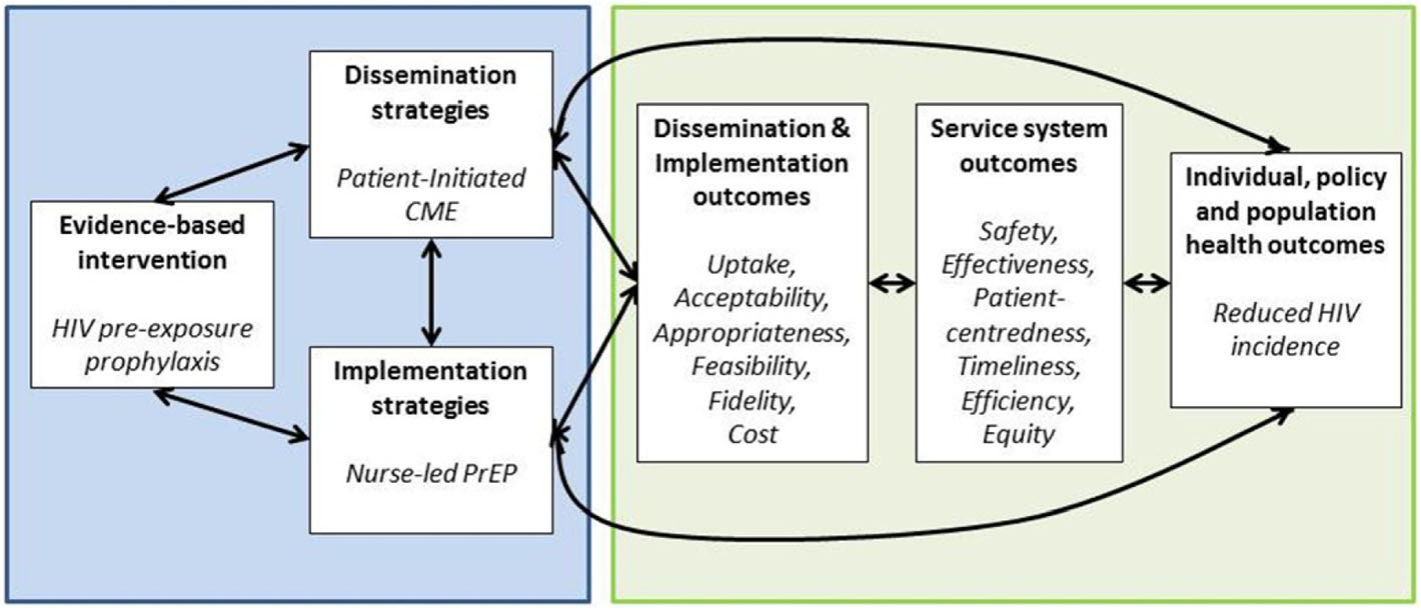
Dissemination & Implementation Framework (adapted from [48])

The sample size for this study is driven entirely by the distribution of the information card/e-card (Step 2 in Fig. 2). Based on prior levels of engagement with a Toronto-based PrEP demonstration project, we conservatively estimate that the 23 CBOs will each distribute 1–2 information cards per month during the 12-month accrual phase of this work, which amounts to approximately 400 distribution events [16]. For our primary analysis, we will calculate the number of PrEP uptake events that occur as a proportion of cards distributed. Our initial estimate of 400 hard copy postcard distribution events will permit estimation of the level of PrEP uptake with a high degree of precision (95% confidence interval of 45–55% for 50% uptake and narrower at any other value) [39].

### Data collection

This study will involve both quantitative and qualitative data sources. The online learning platform hosting our learning modules allows tracking of logins and quiz scores.

The patient baseline questionnaire will cover demographic characteristics, sexual history, sexual behaviour (including validated HIV risk indices), PrEP knowledge and engagement in healthcare. Those with a FP will also evaluate their doctor-patient relationship using a previously tested Physician-Patient Relationship Quality scale addressing general communication, participatory decision-making processes, willingness to recommend their provider to a friend seeking PrEP and overall satisfaction [31]. These domains will be re-assessed at the 6-monthly follow-up questionnaires, with the addition of questions regarding the outcome of their attempts to obtain PrEP (if any) and identified barriers or facilitators to accessing PrEP through this mechanism. Users will enter their unique code to login, allowing linkage of patient and provider data.

Similarly, the physician baseline questionnaire will capture demographic characteristics, practice characteristics, PrEP knowledge, and opinions regarding PrEP. Questions regarding PrEP knowledge and attitudes will be drawn from prior surveys conducted by our group and others, to facilitate comparison between studies [11, 17, 18, 40]. To characterize physician attitudes towards HIV risk behaviours (eg. condomless sex), a modified version of the Healthcare Provider HIV/AIDS Stigma Scale (HPASS) will be administered [41]. These issues will all re-assessed at the 6-monthly follow-up questionnaires, with the addition of questions regarding whether PrEP was initiated in the index patient (and/or others), questions about perceived barriers/facilitators to PrEP prescribing, and repeat assessment of PrEP knowledge.

Prospective data collection from TPH clinic charts will include information on sexual behaviour, laboratory test results and adverse events. The existing TPH appointment scheduling system will be used to track appointments electronically.

We will also obtain qualitative data through a series of separate focus groups for FPs, sexual health clinic nurses/staff, and patients. Focus groups allow for dynamic exchanges between study participants as people share insights from their lived experience and have opportunities to build on one another’s comments. Participants will be drawn from the pool of questionnaire respondents who indicate willingness to join focus groups using a purposive sampling approach. Efforts will be made to include a range of opinions (eg. positive and negative experiences, demographic diversity), based on preliminary analysis of questionnaires. Focus groups will be digitally audiotaped, transcribed verbatim, and reviewed for accuracy. Notes will also be taken during focus groups to capture key insights that emerge regarding group dynamics and key areas of interest. The focus for inquiry will be facilitators and barriers to each component of the two decentralization strategies, including unintended consequences of the approach. We will also explore the perceived impact of the dissemination and implementation interventions on clinician-patient relationships.

A preliminary costing estimate per patient initiated on PrEP will be conducted from the public payer perspective and including both fixed (program) costs and variable (per-individual) costs. We will estimate program costs by estimating additional administration and overhead costs at public health clinics, public health laboratory wholesale purchase costs for test kits and technician time, and costs for the online learning platform. We do not anticipate substantial additional administrative costs at FP offices, since each physician will likely see only a few patients on average. Costs for each individual initiating PrEP will be estimated using a unit cost approach, in which we will estimate the average number of units in each of the following categories and assign each a corresponding cost. Categories include: physician visits for both primary and specialty care (costs from provincial fee schedules), pharmacy administration fees (costs from markup rates), public health costs for nursing staff (costs estimated from labour market rates), and drug costs (costs estimated from publicly available list prices). Because public insurers may negotiate lower drug prices than the list price, we will perform sensitivity analyses across a range of cost deflators. We will also estimate drug costs paid for by patients out-of-pocket and by private insurers. Additional costs will be drawn from the published literature using Canadian data wherever possible.

To estimate the relative contribution of the PICME and nurse-led PrEP strategies to PrEP scale-up in Ontario, we will attempt to quantify PrEP uptake province-wide (ie. estimate the denominator for our project’s numerator) in collaboration with the Applied Epidemiology Unit of the Ontario HIV Treatment Network and Public Health Ontario (facilitated by co-Investigator J. Wilton). For example, records from laboratory test requisitions for all HIV tests conducted in Ontario are housed at Public Health Ontario, and efforts are currently underway to revise the HIV serology requisition to add a “PrEP” checkbox as a reason for testing. Since confirmation of HIV negative status is a core component of PrEP delivery, if added, this requisition change would provide a convenient way to estimate the total number of PrEP users in Ontario (after adjustment for the current non-completion rates of ~ 37% in current ‘reason for testing’ data [M Gilbert, personal communication]). The CME module will include explicit instructions to mark this checkbox on the updated requisitions.

Since a potential unintended consequence of our work may be an increase in specialist referrals, team members running large PrEP clinics in Toronto will quantify referrals received during and 24 month before the study period using clinic administrative data.

### Data analysis

In the primary analysis we will calculate the uptake of PrEP achieved among Toronto MSM using each decentralization strategy (ie. number/proportion of patients initiated on PrEP using postcard distribution as the denominator), broken down according to the various steps in each cascade, as per Table 1. Each step will document the unique code numbers, allowing tracking of each participant through the cascades.

**Table 1:** Cascade of steps involved in PrEP uptake and corresponding data sources

Step	Description	Outcome measure	Data source
2	Card distribution	Number of cards distributed	CBO partners
		Number of e-card impressions	Networking apps
3	Patient initiates module	Number of unique codes used to login	Online learning management platform (see section 5.2)
	Patient completes online patient module	Number of codes/logins reaching end of patient module	
4a	Patient brings postcard to family physician	Number of patients who initiate PrEP discussion with physician	Patient self-report on follow-up questionnaire (section 5.2)
5a	FP initiates CME	Number of unique codes used to login	Online learning management platform
	FP completes CME	Number of codes/logins reaching end of module quiz/certificate	
6a	Physician prescribes PrEP	Number of patients provided PrEP prescription by family physician	Patient and physician follow-up questionnaires
4b	Patient brings postcard to sexual health clinic	Number of patients who approach TPH for an initial PrEP appointment	Patient self-report on follow-up questionnaire
5b	Patient attends sexual health clinic appt.	Number of appointments booked	TPH Appointment Scheduling System
		Number of appointments attended	
6b	Nurse prescribes PrEP	Number of patients provided PrEP prescription by TPH nurse	TPH patient charts

We will use a variety of analyses to assess our secondary objectives. To characterize barriers and facilitators to PrEP uptake in each decentralization strategy, we will use descriptive statistics to summarize questionnaire responses, impact on TPH clinics, and specialist referrals. We will construct two logistic regression models to explore the association between patient and physician characteristics (predictors) with module uptake and with PrEP uptake (outcomes). Key predictors of interest include practice type, identification as an HIV specialist and HIV related stigma for physicians and household income, language, sexual practices and previous STI for patients. We will supplement these quantitative findings with content analysis of open-ended questionnaire responses and transcribed focus group discussions. We will use a coding approach that involves the input of community members through a Community Advisory Board, with the goal of developing a conceptual framework to understand barriers and facilitators of this approach [42].

To assess fidelity we will conduct chart reviews of the sexual health clinic patients to assess adherence to the core components of PrEP delivery, such as clinical indication for PrEP, documentation of HIV seronegativity, correct PrEP prescription, toxicity monitoring, and appropriate STI screening. Because of the impracticality of conducting chart reviews at FP offices, we will rely on indirect measures of fidelity for the PICME strategy data from patient and provider follow-up questionnaires. Such measures will include confirmation of whether modules were completed, follow-up appointments were scheduled following initial presentation of the card, and whether PrEP was ultimately prescribed. We will also explore provider-level correlates of fidelity (eg. practice characteristics, questionnaire responses including HPASS scale,^71^ CME quiz scores) in univariable (chi square) and multivariable (logistic regression) analyses as appropriate.

Finally, we will summarize patient-reported outcomes including satisfaction with clinician-patient relationship using a previously validated Physician-Patient Relationship Quality scale. This scale has demonstrated high levels of internal consistency (Cronbach’s alpha 0.81–0.93) when used in a sample composed primarily of HIV-infected MSM [31].

### Ethics and consent to participate

This study has been approved by the St. Michael’s Hospital (REB #16–348) and Toronto Public Health (REB #2016–16) Research Ethics Boards. An electronic informed consent process will be used for the collection of patient and physician questionnaire data, and additional in-person informed consent processes will be used to obtain consent for the review of sexual health clinic charts and for the focus groups.

### Confidentiality

Information collected during this study will be recorded using the unique identifying code provided to potential participants on the distributed card/e-card. A master linking log, used for the purposes of 6-month follow-up and targeted focus group sampling, will be used to link email addresses to unique identifiers. The log book will be kept locked on site. Only de-identified data will be recorded on all other study documents.

## Discussion

A major impediment to broader PrEP rollout in Canada is the lack of accessible, knowledgeable primary care clinicians who can prescribe PrEP without relying on costly and limited specialists. This implementation science project addresses this challenge, and our findings will inform future PrEP scale-up efforts.

Although FPs have access to large numbers of at-risk, HIV-negative persons, and may have expertise with other components of a combination HIV prevention approach like counselling and addressing substance use and mental health concerns, many may be uncomfortable prescribing PrEP since it involves an antiretroviral drug. The PICME project partly addresses this “purview paradox,” which speaks to the urgent need to build capacity in this group [21, 43].

In addition, this study pilots nurse-led PrEP at two of the four sexual health clinics operated by Toronto Public Health, gathering detailed contextual information on effectiveness and scalability. This data can address some of the challenges involved in nurse-led PrEP, as longitudinal patient follow-up is a departure from the usual model of episodic STI care used in sexual health clinics [44]. From a health policy perspective, and as recommended by the World Health Organization, this intervention represents ‘task-shifting’ [45]. By providing extensive training to the nurses, basing the medical directive on evidence-based Canadian guidelines [19], and ensuring the availability of specialist consultation (as will be provided to FPs), this model is also a form of inter-professional mentorship and collaboration that optimizes the scope of practice of each health professional [26].

By quantifying the impact of the PICME and nurse-led PrEP strategies in our primary analysis, we will ascertain the feasibility and effectiveness of both strategies in increasing PrEP uptake. In addition, we will learn which steps and stakeholders pose the greatest challenges to increased uptake as a means of understand how best to approach future PrEP scale-up. Finally, our costing analyses, in conjunction with our data on clinical outcomes and sexual behaviour, will be used in mathematical models of HIV transmission to evaluate population-level PrEP implementation strategies and impact across Canada.

The study is firmly rooted in the implementation science paradigm outlined in Fig. 2. Using the definition of MacLean, Rabin and others***,*** the proposed ‘PICME’ intervention is a *dissemination* activity - “an active approach of spreading evidence-based interventions to the target audience via determined channels using planned strategies” [46, 47]. Here, PrEP is the evidence-based clinical intervention, FPs represent the well-defined target audience, and patients seeking PrEP are the pre-determined channels who can help disseminate PrEP information using a unique strategy (PICME). Using a definition by Proctor, the proposed nurse-led PrEP intervention is an *implementation* activity, since it involves “the process of putting to use, or integrating, evidence-based interventions within a specific setting” [48].

This study has some limitations. Over the course of the study period, PrEP use may increase outside of PICME or nurse-led intervention, which may result in fewer participants going through the PICME process. However, it may also result in increased interest in guideline-based CME on PrEP. The use of time-series analysis may be done to address such secular trends should they occur. Diffusion effects into the community and among health-care providers may also occur, which may also decrease the number of subsequent participants going through all steps in the PICME process. In addition, more than one patient may present any given FP with the card. While this will decrease the number of linked patient-provider surveys, data on the number of patients approaching a provider will be gleaned through 6-month surveys and focus groups.

Ultimately, the decentralizing of PrEP delivery, when nested in a combination of biomedical and behavioural HIV prevention strategies, has the potential to dramatically decrease HIV incidence. Data from the Kaiser Permanente health system in San Francisco has shown zero new infections since 2012 with increasing PrEP use in a large clinical practice setting serving large numbers of MSM [49]. Similarly, the ‘Demo Project’ observed an extremely low HIV incidence of 0.43 (95%CI = 0.05–1.54) when delivering PrEP in three community-based practice settings in the United States [50]. However, neither of these studies explicitly evaluated knowledge dissemination and implementation outcomes as we will. Our work will gather key data to inform the scale-up of new PrEP dissemination and implementation strategies in the context of our specific target population, healthcare system, and social context.
